# Modelling the Tumour Microenvironment, but What Exactly Do We Mean by “Model”?

**DOI:** 10.3390/cancers15153796

**Published:** 2023-07-26

**Authors:** Constantino Carlos Reyes-Aldasoro

**Affiliations:** Department of Computer Science, City, University of London, London EC1V 0HB, UK; reyes@city.ac.uk

**Keywords:** tumour microenvironment, model organism, in vivo model, in vitro model, mathematical model, computational model

## Abstract

**Simple Summary:**

The word “model” can be used with different meanings and different contexts, like a model student, clay models or a model railway. In some cases, the context can clarify exactly what is meant by “model”, but sometimes several meanings of model can be present in one area. For instance, with reference to cancer research, there can be ambiguity for what is meant by model. This paper reviews the use of the word model as related to cancer research and within the specific area of the microenvironment that surrounds a cancer tumour. The review grouped different definitions of model into four categories (model organisms, in vitro models, mathematical models and computational models) and explored what is meant in each case, mentioning the advantages and disadvantages of the different models Next, a quantitative investigation of the scientific publications listed in the database of the United States National Library of Medicine was performed by counting the frequencies of use of these terms, as well as the components of the microenvironments and the organs modelled with these techniques.

**Abstract:**

The Oxford English Dictionary includes 17 definitions for the word “model” as a noun and another 11 as a verb. Therefore, context is necessary to understand the meaning of the word model. For instance, “model railways” refer to replicas of railways and trains at a smaller scale and a “model student” refers to an exemplary individual. In some cases, a specific context, like cancer research, may not be sufficient to provide one specific meaning for model. Even if the context is narrowed, specifically, to research related to the tumour microenvironment, “model” can be understood in a wide variety of ways, from an animal model to a mathematical expression. This paper presents a review of different “models” of the tumour microenvironment, as grouped by different definitions of the word into four categories: model organisms, in vitro models, mathematical models and computational models. Then, the frequencies of different meanings of the word “model” related to the tumour microenvironment are measured from numbers of entries in the MEDLINE database of the United States National Library of Medicine at the National Institutes of Health. The frequencies of the main components of the microenvironment and the organ-related cancers modelled are also assessed quantitatively with specific keywords. Whilst animal models, particularly xenografts and mouse models, are the most commonly used “models”, the number of these entries has been slowly decreasing. Mathematical models, as well as prognostic and risk models, follow in frequency, and these have been growing in use.

## 1. Introduction

It is now widely accepted that cancer research cannot solely rely on the study of individual cancer cells or a tumour in isolation [1] but rather on the collection of many different cells and their interactions in what is known as the tumour microenvironment [2]. The complex relationships of cancerous cells with healthy cells, immune cells, vasculature, the extracellular matrix, molecules, and other elements that surround and interact with cancer cells are crucial in the development of a tumour and response to treatments [3,4]. The idea of this interaction has been traced back to Stephen Paget who, in 1889, proposed a “seed and soil” theory [5], where cancer cells are the seeds that interact with the organism, the soil, in the spread of tumour cells [6]. Research into the tumour microenvironment, thus, tries to elucidate the mechanisms by which elements such as infiltrating cells [7,8,9], soluble factors [10,11,12], the extracellular matrix [13,14,15] or tumour vasculature [16,17] interact with one another [18]. At the time of writing (July 2023), PubMed, the search engine of the United States National Library of Medicine (NLM) database MEDLINE, returned more than 85,000 entries for the keywords “tumour microenvironment” (https://pubmed.ncbi.nlm.nih.gov/?term=tumour+microenvironment, accessed on 27 June 2023) and slightly fewer, at 82,713, for the same query with tumor instead of tumour (https://pubmed.ncbi.nlm.nih.gov/?term=tumor+microenvironment, accessed on 27 June 2023). Most of these entries have been indexed after 2000.

The direct study of the tumour microenvironment in patients is restricted by the number of patients that are willing to participate in trials; repeated observations are limited, and the uses of new drugs are constrained, needing to have gone through a series of preclinical studies. Histopathology is a useful tool for the study of the tumour microenvironment, but it still has some technical limitations, i.e., it is not possible to visualise blood flow. Therefore, a plethora of indirect methods that overcome these limitations have been developed.

These methods rely on a “model” that, on one hand, simplifies the study of the microenvironment and, on the other hand, resembles it closely, so that any findings can be successfully translated to a patient in a clinical environment. Unfortunately, the concept of “model” is not universally understood. Different disciplines refer to models in distinct ways, which are related to the many definitions of the word itself. The Oxford English Dictionary [19] (https://www.oed.com/search?searchType=dictionary&q=model, accessed on 27 June 2023) includes 17 definitions for the word “model” as a noun and another 11 as a verb, varying from “Something which accurately resembles or represents something else” to “A three-dimensional representation”.

It is, therefore, not surprising that there are many different “models” of the tumour microenvironment. The rest of this work explores some of these definitions as they are understood and used by those who research the tumour microenvironment from different perspectives and assesses the frequencies of use of several keywords related to the tumour microenvironment.

## 2. Different Concepts of Model

### 2.1. Model: “An Animal or Plant to Which Another Bears a Mimetic Resemblance”

Perhaps the most widely used concept of “model” is that related to a model organism: a nonhuman species used for performing experiments that can reveal some understanding of a biological phenomenon [19]. From simple organisms, like the bacteria *Escherichia coli* [20] or yeast like *Saccharomyces cerevisiae* [21], to zebrafish [22], rodents [23] or drosophila [24], model organisms have been extensively used to elucidate anything from aging [25] to Zika [26]. Part of the success of model organisms has been the fact that the operating principles of some cellular processes, like the cell cycle or signalling pathways, are similar in humans and other species that branched out from earlier common ancestors [27]. Rodents have taken a predominant place as a model organism in cancer and other conditions due to several factors: ease of maintenance and transport, high fertility rates, relative low costs and ease of genetic modifications [19]. Specific mouse models can now be used to study perimenopausal depression [28], tuberculosis [29] and myocardial infarction [30], and the genetically engineered mouse is considered by some to be the preferred organism used in cancer studies [31,32]. Cancer can be induced in these models through the administration of a carcinogen [33,34], the diet [35,36] or the transplantation of tissue or cells from patients or cell lines into the model, i.e., xenografts [37,38]. Alternatively, in transgenic animals that have been genetically modified, cancer can occur spontaneously [23,39]. As this type of model is a whole living organism, it is expected that they intrinsically “capture the intricacies of the tumor immune response and microenvironment” [40]. This on its own is one the most important advantages of model organisms, which do not need the design of an environment to model the tumour microenvironment. The organism itself provide the microenvironment from which aspects like therapeutic implications or side effects can be observed [41]. However, there are important shortcomings, as the host organism is a different species than the donor, and there may be a species mismatch between the tumour and the host microenvironments [32,42]. The reliability of the translation from animal models to human diseases, therefore, remains controversial [43,44]. The model then bears a resemblance to the microenvironment of human cancer, but it is not exactly the same.

The tumour microenvironment of a model can be observed through histopathology [45,46,47] and immunohistochemistry [45,48,49], in which tissue is extracted, thinly sliced, and stained with different techniques highlighting important components of the tumour microenvironment, such as macrophages and lymphocytes. An important limitation of histopathology is that there is only one time point of observation. When techniques such as dorsal skin fold window chambers [50] are used, the development of a tumour and its microenvironment can be directly observed through intravital imaging techniques [32,51], which allow repeated observation and the possible effect of treatments [52,53] for a period of time. Alternatively, tissue can be observed using magnetic resonance imaging [54,55] or positron emission tomography [56,57], which are less invasive but have much lower resolution than microscopical techniques.

### 2.2. Model: To Serve or Behave as the Analogue of (A Phenomenon, System, etc.); Or a Three-Dimensional Representation esp. One Showing the Component Parts in Accurate Proportion and Relative Disposition; Or to Produce (a Figure, Likeness, etc.) by Moulding, Carving, etc., esp. in Clay, Wax, or Some Other Malleable Material

Another popular concept of model related to cancer is that of “in vitro” or “in glass” experiments. These models refer to investigations performed with cells, organisms or parts of organisms in Petri dishes or similar equipment and have been used for a long time in cancer-related experiments, such as cell growth [58] and the screening of antitumour substances [59]. These experiments imply artificial conditions and a significant simplification of the microenvironment of a tumour. Conversely, these models offer a number of advantages over in vivo experiments with model organisms, not least the avoidance of animal testing. Advantages of in vitro experiments include lower costs and higher throughput, and they can be considered more amenable to mechanistic analysis [40]. Also, despite the considerable simplification of the environment, these models can have higher human relevance since cancer cells derived from primary patient material can be directly used [60,61]. In vitro models have been considered to have fewer problems with how valid the results for one species are when applied to another species [62]. On the other hand, in vitro models are limited as compared with animal models in the complexity they can offer. There is no physiological response, and it is more difficult to observe side effects.

A simple setting to mimic the tumour microenvironment is to co-culture cancer cells with cells of the tumour microenvironment, like myofibroblasts [63], cancer-associated fibroblasts [64], endothelial cells [65] or stromal cell types and/or the extracellular matrix [66]. These co-cultures can then be used to perform a wide variety of experiments related to cell proliferation [67], migration [68,69], invasion [70] or treatment and drug combinations [71,72]. Despite the simplicity of these experiments, the inherent 2D nature of the cultures is a major limitation, as the interactions between cells and the environment do not resemble the 3D nature of a tumour and its microenvironment [73,74]. Accordingly, 3D in vitro models of the tumour microenvironment have evolved significantly, for instance, in breast cancer [75] and now include multicellular aggregates, like spheroids [76,77] or organoids [78,79], which are maintained in different settings, such as purified extracellular matrix gels, hanging drop cultures, and 3D gels or 3D scaffolds [80] of meshes or sponges, which offer a greater number of conditions, such as porosity, biodegradability, chemical composition, transparency, etc. [74]. A further complexity can be introduced to in vitro models by allowing external interaction, thus simulating metabolic processes [81], or providing complex geometries, such as branching structures, that mimic the vasculature of a tumour [82]. These models are known by different names: 3D bioprinted, microfluidic, tumour-on-a-chip or organ-on-a-chip [83,84,85,86,87,88]. One of the major advantages of these models over animal models is the observation, as the settings themselves are easy to examine with microscopes or in other settings.

### 2.3. Model: A Simplified or Idealised Description or Conception of a Particular System, Situation, or Process, Often in Mathematical Terms

A mathematical model can be understood as the simplification and abstraction of a complex phenomenon and its subsequent description in mathematical equations. A model should tackle one or more biological or clinical hypotheses and analyse experimental data together with the formulation of a mathematical description, i.e., the model itself, and undergoes a cycle of refinements until it can be validated [89,90].

A classic example of a mathematical model is the Malthusian growth model [91] that assumes that a population (P_0_) grows in time (t) in an exponential way depending on the growth rate (r) following the equation P(t) = P_0_ e^rt^. This model is similar to the cancer initiation model proposed by Armitage and Doll [92] describing the incidence rate (I) of a cancer at age t as I(t) = k t^n^, where k is a constant, and *n* is the number of stages (or mutations) that must be passed for a cell to become malignant. These two models are descriptive models, i.e., they describe the broad characteristics of a phenomenon or can be used to predict or prognosticate a future state. When the description refers to the time of occurrence of an event being modelled, the process is sometimes called a survival analysis [93]. If the model takes into account one factor (time) but ignores other factors (such as ethnic group, age or lifestyle), the model is considered univariate [94]. Multivariate statistical models [95], on the other hand, consider several variables at the same time, for instance, the correlation between the overall survival of patients with non-small-cell lung cancer and the concentrations of amino acids and metabolites measured from blood samples [96].

Alternative to descriptive models are those considered mechanistic or conceptual [97], which attempt to explain the processes that drive phenomena [98] and from which it is possible to derive biologically important characteristics of a tumour, for instance, that distal recurrence of glioblastoma depends on a hypoxic microenvironment and the migration and proliferation rates of tumour cells [99].

Models that provide the same results every time are considered deterministic, and those which include a certain randomness in the process are considered stochastic [97]. Stochastic models of the tumour microenvironment [100,101,102] are more common than deterministic ones [103] by an approximate ratio of 10 to 1, which is probably a reflection of the many factors related to cancer, like somatic evolution, which are not deterministic [104].

The scale, or point of view, of a model, provides different resolutions at which the model operates: at an organ scale, they are considered macroscale models [105] and, at the cell level, they are considered microscale models [106]. Intermediate scales are sometimes referred as mesoscale models [107] and are related to mesoscale imaging [108], which aims to link the information of cells, organs and tissues. Some authors consider that there is a gap at the mesoscale, for instance, to relate interactions of cells that are far away from each other [109]. The term mesoscale itself originated in meteorology as an intermediate scale between large- and small-scale systems. The nature of the tumour microenvironment can be studied at different scales at the same time; thus, many models are considered “multiscale” [110,111,112,113,114,115], as they consider, from molecules to cells to tissue-level phenomena [116,117], how the extracellular matrix is altered [118,119], or an avascular tumour growth and cell model [120]. It is important to consider that any model should be able to reproduce data that have been observed through experiments [121] and, as such, models at different scales require validation at different scales as well [122]. Some authors stress the importance of incorporating cellular models into whole-organ models [123]. This can be an advantage of mathematical models over in vitro models, and it is one that in vivo models provide intrinsically.

An interesting perspective to formulate models is to consider the cell as a basic unit, i.e., a virtual cell [124,125], with a set of rules for behaviour. The unit is sometimes called an “agent”, with rules to proliferate, reproduce or transform depending on interactions with its external microenvironment [111] and probabilistic rules [126]. Different types of cells (tumour, immune or dendritic) constitute different agents [127]. Since these approaches build a study up from single cells, they are considered “bottom-up” [128]. “Top-down” approaches, on the other hand, zoom out and focus on whole organs or consider cells as a group or population. The behaviour is considered as a mean of all the cells and not as individuals [122]. It is possible, of course, to start not at the top or the bottom, but rather somewhere in between with “middle-out” models [129,130,131]. A middle-out model is useful in cases where there are rich levels of biological data than can be used as a starting point from which to reach up and down [123], or when the phenomena to be modelled are themselves in the mesoscale, like microcirculation [132].

In an alternative approach, these cells, whether cancerous or healthy, can be considered as species that strive for survival, treating cancer as a problem of ecology and evolution [133,134,135] and considering subpopulations within a single cancer [136]. The ecological and evolutionary perspectives can themselves be intrinsically related to cancer, as has been proposed by several authors [137,138,139]. An example of this approach is the branching process [140,141] in which, as time passes, a cell may divide, die or mutate at certain rates. After a number of cycles, mutations may accumulate in the population of cells. From a simple formulation like this one, it is possible then to significantly increase complexity by adding different types of cells, i.e., cells of the immune system [142]. As such, models have now been proposed for migration [143], tumour growth [144], invasion [145], angiogenesis [146,147], treatment and recurrence [148], cancer cell intravasation [149], fluid transport in vascularised tumours [150], macrophage infiltration [151], response to radiotherapy [152] and optimisation of chemotherapy [153]. For reviews of mathematical modelling of cancer, the reader is referred to [89,90,98].

As many of the previously mentioned approaches require computer simulations, these models are sometimes called in silico models or computational models. Some mathematical models are purely mathematical, like that of Armitage and Doll, which does not require simulations or computations but merely applies an equation. However, many mathematical models apply numerical methods and are intrinsically computational [154]. Some authors [155] distinguish mathematical models when they use a continuous model using mathematical equations from computational models, which are discrete and based on a series of steps or instructions. Still, in many cases, distinctions between mathematical and computational are not considered, and some authors use the terms “mathematical model” and “computational model” interchangeably [156], and others consider a model itself to be both mathematical and computational [97,157,158,159,160,161]. For more information about mathematical and computational models of the tumour microenvironment and cancer, the reader is referred to [97,112,122,162,163].

Mathematical and computational models include numerous advantages: no need of animals or tissues, lower costs and the rapidity at which simulations can be generated. However, the limitations are numerous, and not the least is the inherent simplicity of any mathematical model as compared with a living organism, a complex disease like cancer and a complex setting like the tumour microenvironment.

### 2.4. Model: To Devise a (Usually Mathematical) Model or Simplified Description of (a Phenomenon, System, etc.)

Despite the close relationship between the mathematical and computational approaches, there are different methodologies that are fundamentally computational. In these cases, computational methods are applied to process, analyse and extract information from datasets. As opposed to a “model” that describes the growth of a tumour, these methods can, for instance, count something [164] or measure colour [165]. What is modelled is not the cells or the cancer itself, but rather derived features, like the shape of a cell or a vessel [72], the movement of cells or fluorescent intensity. There does not need to exist an underlying mathematical abstraction of cancer or a biological process in these methodologies, but the information extracted relates to conditions of the cancer, like the cellularity [166].

Computational methods that belong to areas of computer vision, image processing, machine learning and, more recently, deep learning can be applied. Features related to important characteristics, like the number of nuclei identification [167] or microvessel density [168], can be extracted. Surely, these computational methods can extract features or quantities that can be then used to inform mathematical models. For instance, to estimate vascular permeability [53] in tumours, the fluorescence intensity can be acquired, and then, through image-processing techniques, the vasculature can be segmented, the intensity inside and outside the vessels calculated, and these quantities fed to the Patlak Model [169] to model blood extravasation. The effect of vascular disrupting agents on tumours can be assessed using the velocity of red blood cells travelling inside a tumour, and a model of movement can be applied to measure the velocity of the cells [170]. The spatial heterogeneity in a tumour microenvironment [171] can be assessed by identifying and mapping cells from histological samples, and then ecological models can be used for the information extracted.

To complicate matters, another quite different computational type of model has been gaining popularity. Namely, those are models associated with the areas of artificial intelligence, artificial neural networks and deep learning. These models are inspired by neurobiology and the simplification of a neurone as a unit with many input signals, which are weighted, i.e., multiplied by individual values, and then combined (i.e., summed) to produce a single or multiple output value. This model is known as the McCulloch–Pitts model of a neuron [172,173]. Many neurones, sometimes also called nodes or units, with this and many other functions, are then combined into layers with a specific structure, sometimes called an architecture. With time and increase in computer power, these models of artificial neural networks increase in complexity, adding more and more layers with millions of neurones to their architectures and, thus, gaining the name “deep”. One key difference is that, unlike other mathematical or computational models in which fine-tuning of the parameters is performed manually by a person (hand-crafted), these have huge numbers of parameters that self-tune when presented with a large amount of training data, i.e., raw data, like an image coupled with class labels that indicate what is where. This process through which the parameters of the architecture adapt is called “learning”, and the area in general is known as machine learning and, in particular, deep learning for larger architectures. Thus, a specific model can be equally used to analyse images of cats and dogs or images of the tumour microenvironment depending on the training data that are provided. Sometimes the arrangement of the basic blocks or structure is called architecture and, once it is specifically trained for a task, it is called a model, but as in other cases, architecture and model are used interchangeably. These models are normally known by short acronyms, like CNN (for convolutional neural network) of VGG [174] (after the Visual Geometry Group at Oxford University), sometimes followed by numbers associated with the number of layers of the architecture like VGG16, as well as AlexNet [175] (after the name of the designer of the architecture Alex Krizhevsky), U-Net [176] (after the shape of the architecture, like a letter U), or GoogLeNet [177] (after the affiliation of some of the authors where the architecture was introduced). For introductory reviews to deep learning, the reader is referred to [178] and, for neural networks and deep learning for biologists, to [179]. For more specific reviews on deep learning applied to cancer and histopathology, the reader is referred to [180,181,182,183,184,185]. The following paragraph illustrates with a few examples how deep-learning models are applied.

The differences between a breast stromal microenvironment and benign biopsies in haematoxylin and eosin (H&E) slides were distinguished using a VGG model [186]. The model was then used with a different dataset to detect a higher amount of tumour-associated stroma in ductal carcinoma in situ for grade 3 compared with grade 1. Cancer grading was calculated from prostate cancer H&E slides with a combination of several CNNs that performed detection and classification and, for tissue, with a posterior slide-level analysis, which provided a Gleason grade [187]. Patient survival was predicted from colorectal histology slides [188] by applying a VGG19 model for the classification of the slides into a series of classes (adipose, background, debris, lymphocytes, mucus, smooth, etc.) from which a combination of values was used to create a “deep stromal score” with considerable prognostic power, especially for advanced cancer stages. In another study [189], patient survival was predicted from a score (tumour-associated stroma-infiltrating lymphocytes (TASIL) score), which was calculated from spatial co-occurrence statistics (stroma–stroma, stroma–lymphocyte, etc.) that were extracted using a DenseNet model [190] to segment each class in head and neck squamous cell carcinoma H&E slides.

## 3. Quantitative Evaluation of the Presence of Different Models in Medline

To assess the distribution of the different definitions of the word model as related to the tumour microenvironment, a quantitative and unbiased analysis was performed. The analysis mined the MEDLINE database of the United States National Library of Medicine at the National Institutes of Health. Mining was performed using the PubMed search engine through a series of queries with combinations of keywords and basic terms as previously described [191] with custom scripts written in Matlab^®^ (The Mathworks^TM^, Natick, MA, USA) and available at https://github.com/reyesaldasoro/TumourMicroenvironmentModels, accessed on 27 June 2023. The basic terms were the search URL of PubMed (https://www.ncbi.nlm.nih.gov/pubmed/?term=, accessed on 27 June 2023) and tumour microenvironment in British and American spellings ((“tumor microenvironment”) OR (“tumour microenvironment”)), and “cancer microenvironment” was also included with an OR. Dates were restricted to 2000–2023 (2000:2023[dp]). The keywords were manually curated based on the previously described definitions of the word model and are shown in Table 1. The concatenation was performed sequentially with one keyword at a time.

The following caveats should be considered when observing the results. A single entry could be retrieved more than once, e.g., “Imaging interactions between macrophages and tumour cells that are involved in metastasis in vivo and in vitro” was counted for both in vivo and in vitro. Similarly, the same type of model could be referred to with two different keywords, like mouse and mice. The entries were mined if the keyword appeared in the PubMed record, which included title, abstract and Mesh terms. That is, if the keywords only appeared in the main text of a paper, it was not retrieved. Furthermore, it is very important to note that the term tumour/tumor could include benign tumours and, thus, the results were not restricted to cancer.

**Figure 1:**
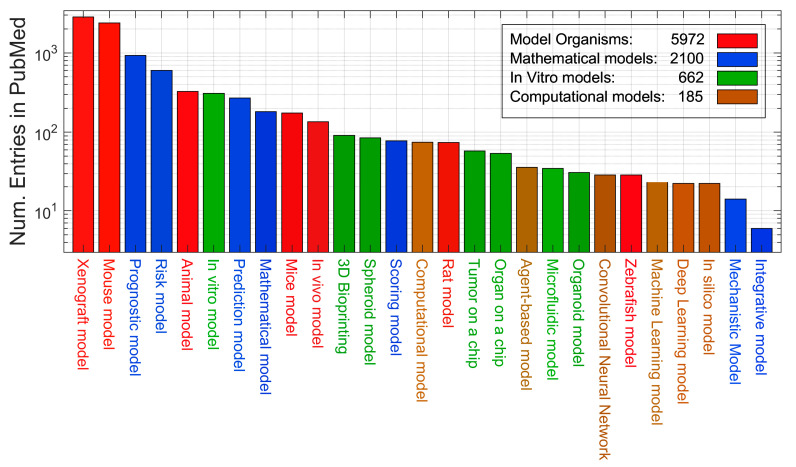
Numbers of entries indexed in PubMed for individual queries. Each query concatenated the individual keyword with the date range of (2000:2023[dp]) and restrictions corresponding to tumour microenvironment (((“tumor microenvironment”) OR (“tumour microenvironment”)) OR (“cancer microenvironment”)). Colours are allocated for organism (red), mathematical (blue), in vitro (green) and computational (brown) models for visualisation purposes. The legend in the top right indicates the aggregates per group.

**Figure 2:**
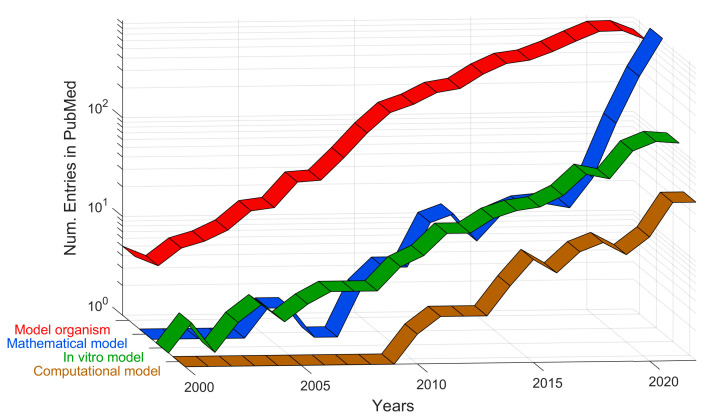
Numbers of entries indexed in PubMed for individual queries per year of publication aggregated by the four groups. Each coloured ribbon corresponds to the sum of the keywords of each group, and the year increases as indicated by the axes on the right. It should be noticed how some techniques are more established (i.e., organism model) whilst others are more recent (computational model).

The total number of entries in PubMed for each of the keywords is shown in Figure 1 as a bar chart. Colours are used to group each keyword according to the definitions of model. In Figure 2, the entries are aggregated into four groups and are shown per year as ribbons with the same colours as in Figure 1.

The first observation is that the most frequent entries for the tumour microenvironment were those related to animal models, far more than the in vitro models. Since the scale of the vertical axis was logarithmic, xenograft and mouse were an order of magnitude above most other keywords. These were followed by the mathematical keywords of prognostic and risk. Despite the simplicity of in vitro models and the perceived lack of human relevance of animal models, these latter ones dominated the research on the tumour microenvironment. However, the temporal trends shown in Figure 2 show that there was a slight decrease in the number of entries related to animal models in the last 3–4 years. Furthermore, whilst the term mathematical model appeared much more recently than the organism or in vitro models, the growth was faster and overtook both, especially in the past 5 years. The term computational model appeared later but also showed an increasing trend, although not as high as for mathematical model. It will be interesting to observe these trends in future years.

**Figure 3:**
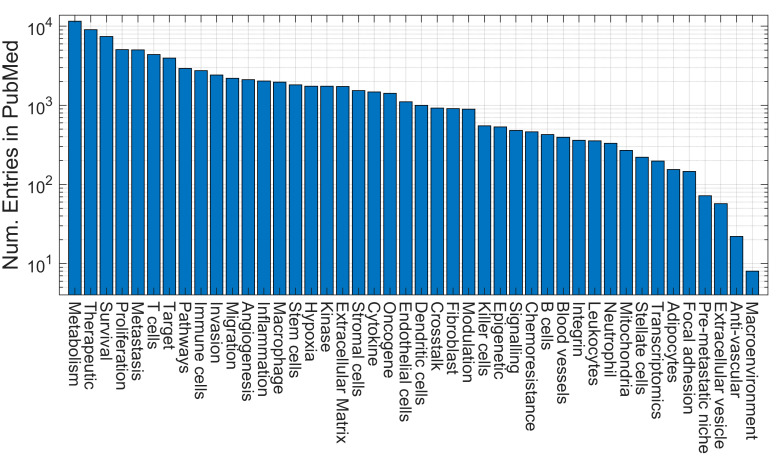
Numbers of entries indexed in PubMed for individual queries. In this case, the query included a keyword, e.g., angiogenesis, and the word “model”. It should be noted that the vertical axis is logarithmic.

**Figure 4:**
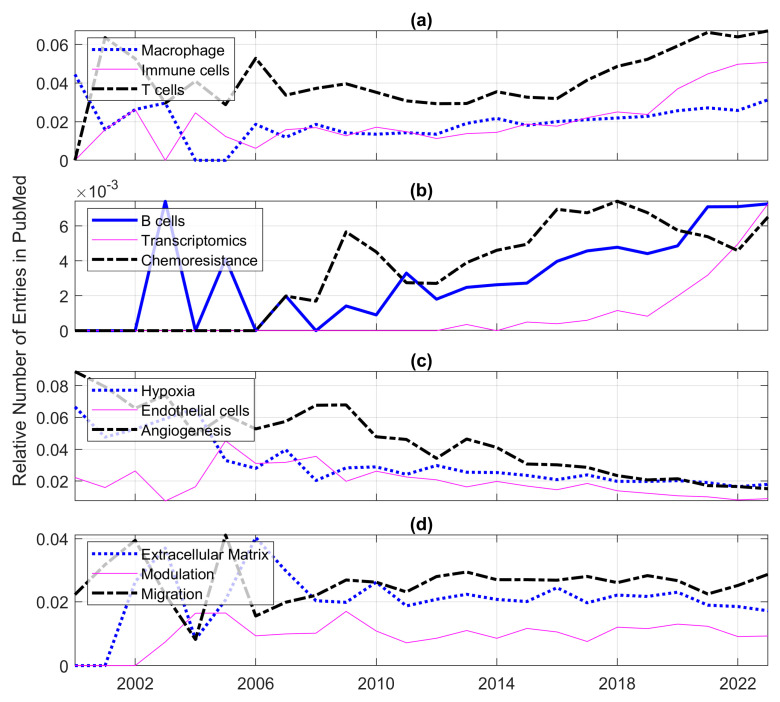
Relative numbers of entries indexed in PubMed for individual queries. Each ratio represents the entries of one keyword to the total number of entries of all the keywords per year. (**a**) Keywords that showed the largest increases. (**b**) Keywords that showed increases but at a smaller scale (notice the differences in the vertical axes). (**c**) Keywords that showed decreased. (**d**) Keywords that remained relatively stable.

Next, to identify important components of the microenvironment and their frequencies of appearance in PubMed, 39 keywords related to the microenvironment (e.g., T cells, endothelial cells, B cells, invasion, metastasis, inflammation, cytokine, pathways, etc.) were added to the queries (e.g., (“cytokine”) AND ((“tumor microenvironment”) OR (“tumour microenvironment”) OR (“cancer microenvironment”) AND (model) AND (2000:2023[dp])). Figure 3 shows the frequencies of appearance of the keywords in decreasing order, starting with metabolism, therapeutic and survival and decreasing towards pre-metastatic niche, extracellular vesicle, anti-vascular and macroenvironment. Again, in addition to the caveats previously mentioned, it should be taken into consideration that this figure indicates only how frequently the terms appeared in the query. For instance, the frequency of the term neutrophils was one order of magnitude lower than the term macrophages. Still, the most common term and possibly the related research question was to investigate the metabolism of the tumour microenvironment. To investigate the trends of these terms with time, a relative count of the keywords per year was performed. The number of entries of each keyword was divided by the total number of entries of all the keywords per year. As could be expected, the uses of certain keywords increased, others decreased and some remained relatively constant. Figure 4 shows some selected keywords, showing these trends, e.g., whilst T-cells increased (Figure 4a), angiogenesis decreased (Figure 4c) and extracellular matrix remained stable (Figure 4d). Some keywords, like B-cells, transcriptomics or chemoresistance, also grew, but the numbers of entries were much smaller than others, so these were shown separately (Figure 4b).

**Figure 5:**
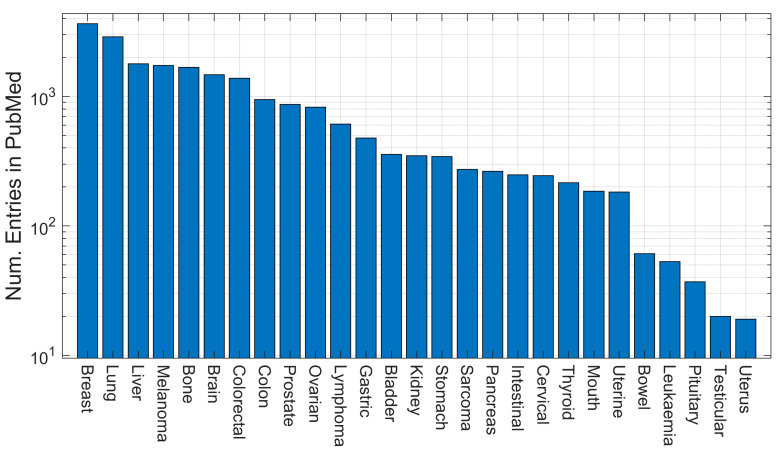
Numbers of entries indexed in PubMed for individual queries. In this case, the query included organ-specific keywords.

Organ-specific keywords were used to investigate the most frequently modelled microenvironments, and the results are shown in Figure 5. Breast and lung were the most common terms, followed by liver, melanoma and bone, with very similar numbers of entries. These numbers partially correlated with the incidence and mortality rates of cancer. Worldwide, the most common cancers by incidence are breast, lung, colorectal, prostate and stomach and, by mortality, are lung, colorectal, liver, stomach and breast [192]. Proportionally, melanoma, bone and brain were more common in research entries in PubMed than their corresponding incidence and mortality rates. At the bottom of the list were bowel, leukaemia, pituitary, testicular and uterus. It is interesting that some related terms, like colorectal/colon, could have similar numbers of entries whilst others, like uterine/uterus, could be orders of magnitude different.

**Figure 6:**
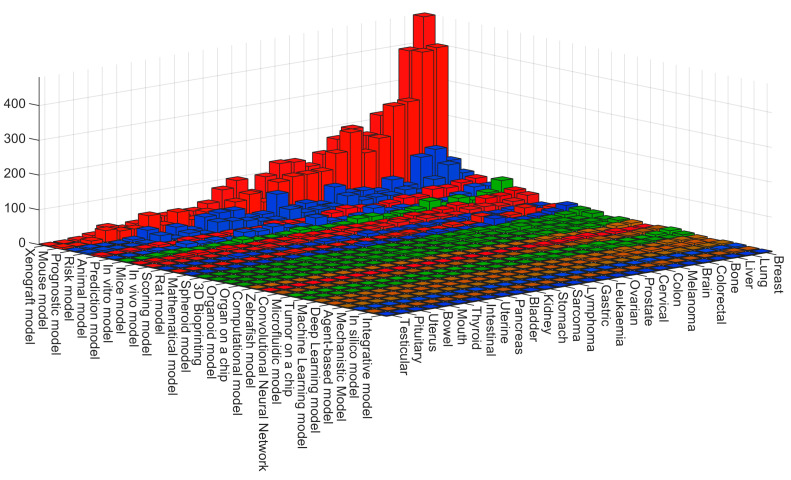
Numbers of entries indexed in PubMed for queries with pairs of keywords for models and organs. Each 3D bar corresponds to the number of entries in PubMed of a specific pair of keywords. The colours correspond to those of Figure 1.

**Figure 7:**
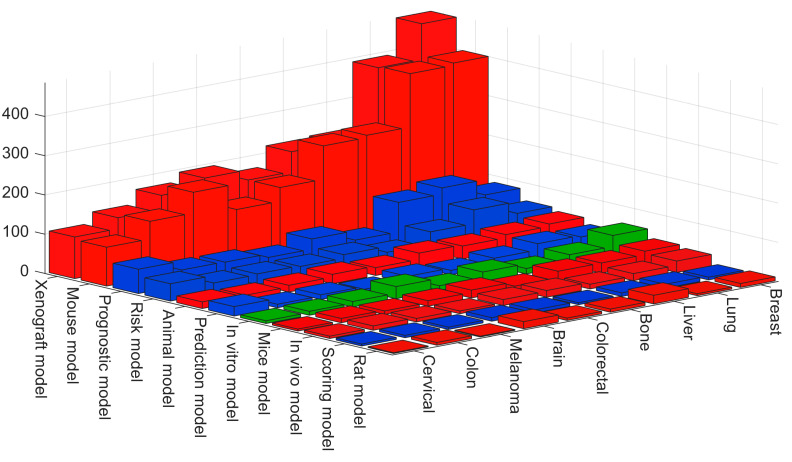
Numbers of entries indexed in PubMed for queries with pairs of keywords for models and organs for cases with the highest results from Figure 5.

A combination of keywords for the models and the organs as pairs (e.g., “(xenograft) AND (brain)” added to the query) is displayed in Figure 6, and a magnified view the terms with most results is shown in Figure 7. These figures show that the most common combination was xenograft with breast with 479 entries, followed closely by mouse model-breast (398), mouse model-lung (392) and xenograft-lung (388). The most frequent entries when using prognostic or risk models were lung, liver, breast, cervical and colorectal. For in vitro, the most frequent entries were breast, brain, lung and bone.

## 4. Conclusions

Whilst the most common setting to investigate the tumour microenvironment is model organisms, recent years have shown a slight decrease in the number of entries in PubMed. In vitro models also showed growth with a slowdown in the last 2 years of the analysis here presented. On the other hand, the number of entries using mathematical models grew steadily and are now as common as the number of entries for in vivo models. The use of computational models also grew, especially agent-based models and convolutional neural networks. It will be interesting to see how these trends continue in the near future.

The basic idea behind the models here described is that these constitute a simplified, idealised and more accessible representation of something more complex and hard to observe, in this case the tumour microenvironment. Whilst it should always be well understood that no model is a perfect representation of reality, a good model should capture some essential characteristics of the microenvironment and permit successful experimentation from which observations can be translated to patient treatment or care. It should always be considered that not everyone understands models in the same way; thus, it is important to make an effort to use these terms in ways that avoid confusion, if possible. For instance, when talking about deep learning, the term “architecture” could be used instead of model. Adding the word “organism” in cases of animal models could also help, e.g., “the mouse has become the favorite mammalian model *organism*”. Similarly, in mathematical cases, the specification of a risk model or a mechanistic-model and not just a model would improve clarity. Biologically and clinically, there are still many unanswered questions related to the tumour microenvironment and all its components. Interdisciplinary research related to the microenvironment is growing, and as such, a single study may include, say, in vitro models that are then processed with deep-learning models or histopathology slides that are analysed with machine-learning models that then feed a prognostic model of survival. Therefore, a clear understanding of what is meant each time that the word “model” appears in a paper is necessary, and researchers from all sides of the spectrum should bear in mind that not everyone understands the same meaning from “model”.

## Figures and Tables

**Table 1:** Keywords used to create queries to mine PubMed grouped according to the four definitions of model.

Definition	Keywords
Model organism	Animal model. Mouse model. Mice model. Rat model. Zebrafish model. Xenograft model. In vivo model.
In vitro model	In vitro model. Tumor on a chip. Microfluidic model. 3D Bioprinting. 3D model. Organoid model. Spheroid model. Organ on a chip.
Mathematical model	Mechanistic Model. Scoring model. Prediction model. Risk model. Integrative model. Mathematical model. Prognostic model.
Computational model	In silico model. Computational model. Deep Learning model. Machine Learning model. Convolutional Neural Network. Agent-based model.
